# Prognostic Significance of P16^INK4a^ Expression in Penile Squamous Cell Carcinoma: A Meta-Analysis with Trial Sequential Analysis

**DOI:** 10.1155/2018/8345893

**Published:** 2018-07-19

**Authors:** Jiayi Zhang, Hengcheng Zhang, Yanyan Xiu, Hong Cheng, Min Gu, Ninghong Song

**Affiliations:** ^1^Department of Urology, The First Affiliated Hospital with Nanjing Medical University, Nanjing 210029, China; ^2^Department of Dermatology, The First Affiliated Hospital with Nanjing Medical University, Nanjing 210029, China

## Abstract

**Background:**

Recently, P16^INK4a^ expression has been shown to be correlated with cancer-specific survival (CSS) in penile squamous cell carcinoma (SCC). Therefore, we performed this meta-analysis to clarify the prognostic value of P16^INK4a^ for penile SCC.

**Methods:**

A systematic search was performed in PubMed, Embase, and Web of Science to identify all relevant articles up to May 25, 2017. Hazard ratios (HRs) and corresponding 95% confidence intervals (CIs) of included studies were pooled to estimate the prognostic value. Trial sequential analysis (TSA) was performed to assess the quantity and strength of survival evidence.

**Results:**

Five retrospective studies consisting of 323 cases were finally included. We found that P16^INK4a^ positive expression was significantly associated with a better CSS of penile SCC (HR=0.45, 95%CI: 0.30-0.67, P<0.001). No heterogeneity or publication bias was noted among the included studies. Furthermore, TSA demonstrated that the findings were based on sufficient evidence.

**Conclusions:**

P16^INK4a^ positive expression is independently associated with improved CSS for patients with penile SCC.

## 1. Introduction

Penile cancer is an uncommon male cancer, with a very low incidence in Western countries (under 1/100 000 men in the United States and 0.1-0.9/100 000 men in Europe). However, its prevalence in the developing countries of Africa, Asia, and Latin America is greater (up to 3.7/100 000 men), and the cancer-related mortality remains significant in these areas [1, 2]. The differences in prevalence are likely explained by the variance in risk factors such as human papillomavirus (HPV) infection, living in rural regions, poor genital hygiene habits, and the practice of neonatal circumcision [3]. HPV infection has been demonstrated to play an important role in the pathogenesis of penile cancer and is associated with certain histological subtypes, in particularly squamous cell carcinoma (SCC), the most common histological subtype of penile cancer [4, 5].

In recent years, overexpression of P16^INK4a^ has been reported in HPV-related tumors such as the head and neck SCC and cervical cancer [6], which is considered a reliable marker for the presence of high-risk HPV DNA. However, limited data are available regarding P16^INK4a^ detection and its prognostic significance in penile SCC. P16^INK4a^, known as a tumor suppressor, is shown to induce cell cycle arrest and prevent cell division by cyclin-dependent kinase 4 (CDK4) and cyclin-dependent kinase 6 (CDK6) inhibition, so as to control disease progression by mediating G1 arrest [7]. Besides, P16^INK4a^ is involved in retinoblastoma (Rb) inactivation by the viral E7 protein [8]. Recent studies reported deregulated P16^INK4a^ expression in different populations with penile SCC, and P16^INK4a^ expression has been shown to be correlated with an improved cancer-specific survival (CSS) in penile SCC [1, 9–12]. These evidences support the hypothesis that P16^INK4a^ might be a prognostic marker for patients with penile SCC, which has not been conducted for a meta-analysis to date. Therefore, we searched relevant researches and performed this quantitative meta-analysis to clarify the prognostic value of P16^INK4a^ expression in penile cancer.

## 2. Materials and Methods

This meta-analysis was conducted according to the guidelines of the PreferredReporting Items for Systematic Reviews and Meta-Analyses (PRISMA) [13].

### 2.1. Search Strategy

A systematic literature search was performed in the electronic databases PubMed, Embase, and Web of Science to identify all relevant articles up to May 25, 2017. We used the combinations of the following keywords: (‘P16' or ‘P16^INK4a^'), and ‘penile' and (‘cancer' or ‘carcinoma' or ‘tumor' or ‘neoplasm'), and (‘survival' or ‘prognosis'). The reference lists and bibliographies of all the eligible studies were, respectively, checked for other eligible investigations.

### 2.2. Inclusion Criteria

**Figure 1:**
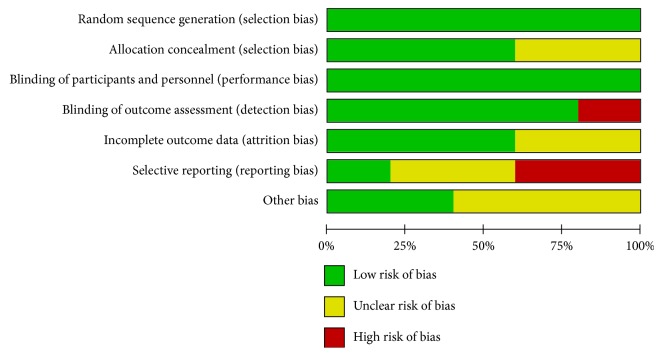
Risk of bias graph about each risk of bias item presented as percentages across all included studies.

**Figure 2:**
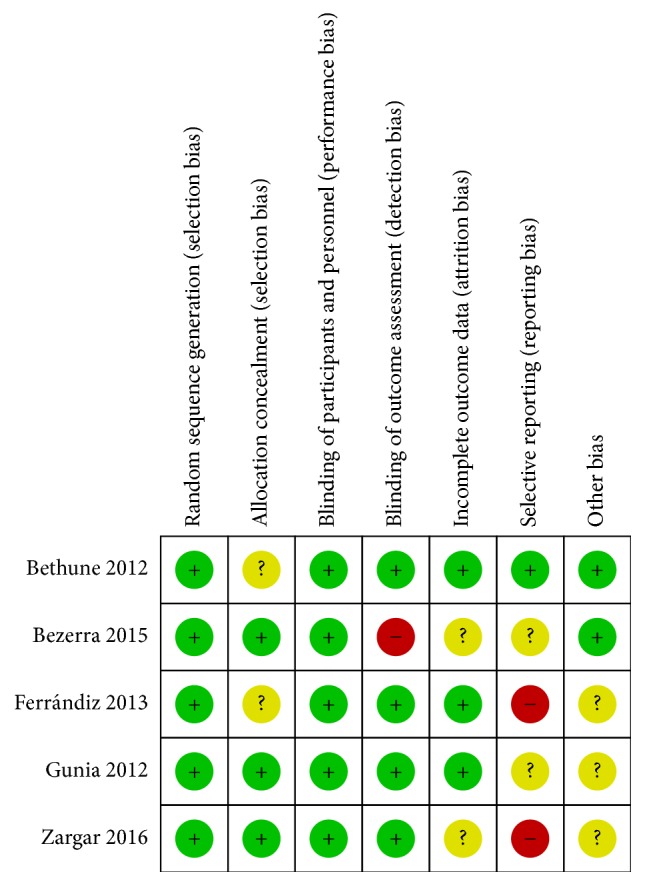
Risk of bias summary graph about each risk of bias item for each included study.

**Figure 3:**
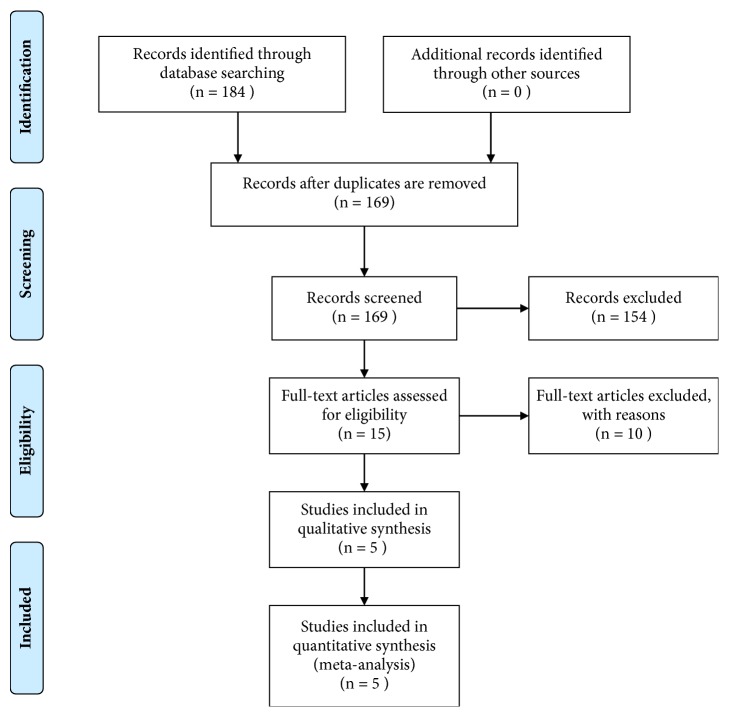
PRISMA flow diagram of study selection and search strategy.

Studies were considered eligible if they satisfied the following criteria: (1) they are English publications; (2) the association of P16^INK4a^ with the prognostic value in penile cancer has been described; (3) P16^INK4a^ expression has been detected by immunohistochemistry (IHC); (4) studies reported cancer-specific survival (CSS) with hazard ratios (HRs) and 95% confidence intervals (CIs). Only research studies were considered eligible for the meta-analysis. Letter, brief communication, case report, and review were excluded according to our criteria. If more than one article on the same series of study subjects had been published, the most recent study with comprehensive data was selected. We also searched for relevant publications in Chinese (http://www.cnki.net/) to comprehensively understand the role of P16^INK4a^ in penile SCC. Moreover, we used the Cochrane Risk of Bias tool and graded each potential source of bias as a low, high, or unclear risk (http://www.cochrane.org/; Figures 1 and 2). A flow diagram with details of the study selection process is presented in Figure 3.

### 2.3. Data Extraction

All eligible studies were reviewed according to the inclusion criteria by two independent reviewers (JYZ and HCZ), and uncertain data were reassessed by NHS. The following elements were extracted from each literature: (1) the first author and publication year, (2) characteristics of the studied population, (3) the number of patients, (4) detection method of P16^INK4a^ and cut-off definition, (5) follow-up time, and (6) HRs along with their 95% CIs and P values. If HRs and 95% CIs were not directly reported in publications, survival data were extracted from Kaplan-Meier (K-M) curves by using previously described methods [14, 15]. Data from graphical survival plots were independently evaluated by two researchers (JYZ and HCZ) using Engauge Digitizer v.5.1 (license type: GPL, developed by Mark Mitch; Category: C:∖Science/CAD). All the above-mentioned data are presented with details in Table 1.

### 2.4. Statistical Analysis

In the present meta-analysis, HRs and corresponding 95% CIs of included studies were combined to calculate the prognostic value of P16^INK4a^ in penile SCC. A pooled HR of <1.0 represented an improved prognosis, and an HR of >1.0 correlated with poorer prognosis. Heterogeneity among studies was identified by the Cochran Q test and was quantified by the Higgins I^2^ statistic. Quantification of heterogeneity has been assigned low, moderate, and high to I^2^ values of 25%, 50%, and 75%. Besides, Galbraith plot and sensitivity analysis for included studies were, respectively, implemented to identify the source of heterogeneity. The random-effects model was used when significant heterogeneity was observed (P<0.10 or I^2^> 50%); otherwise, the fixed-effects model was applied [16]. Publication bias was assessed by funnel plot visual inspection and was statistically evaluated by Begg's test and Egger's test, and P<0.05 was considered statistically significant. All above-mentioned statistical analyses were performed by Stata V.12.0 (StataCorp LP, College Station, Texas, USA) and Microsoft Excel (v.2010, Microsoft Corporation, Redmond, Washington, USA).

### 2.5. Trial Sequential Analysis

Meta-analyses may result in type I errors due to random error from the studies included in the meta-analysis which had a small sample size, publication bias, and low quality, and studies whose conclusions tended to be changed by later studies with a larger sample size [17]. We did trial sequential analysis (TSA) for final included studies in the meta-analysis to estimate and correct these limitations and determine whether cumulative evidence is enough reliable. Our assumptions included two-sided testing with a type I error of 5%, and a type II error of 20% (power of 80%). The main results of TSA were showed in a cumulative Z-curve graph, and the monitoring boundary of required information size in the graph was determined according to O'Brien-Fleming *α* spending function [18]. Besides, the futility boundary was set on the basis of O'Brien-Fleming *β*-spending function. When the cumulative Z-curve crosses the trial sequential monitoring boundary or enters the futility area, a sufficient level of evidence may have been reached, and no further trials are needed. If the cumulative Z-curve does not cross any of the boundaries, and the required information size has not been reached, there is insufficient evidence to reach a conclusion. TSA was carried out by the statistical software, TSA version 0.9 beta (User Manual for TSA, Copenhagen Trial Unit 2011, http://www.ctu.dk/tsa).

## 3. Results

### 3.1. Eligible Trials

As shown in Figure 3, a total of 184 articles were identified through the primary comprehensive online search. Then 169 records were screened by title and abstract, after 15 duplicates were removed. Furthermore, 154 studies were excluded by preliminary review, and 10 studies with insufficient survival data were considered ineligible by full-text screening. Finally, a total of five retrospective studies were included in the meta-analysis [1, 9–12].

The main characteristics of the included studies are summarized in Table 1. A total of 323 participants from Canada, Germany, Spain, and the United States were enrolled, with the numbers of cases of P16^INK4a^ positive and P16^INK4a^ negative. The mean or median age of included patients ranged from 60.0 to 69.0 years. All of the studies performed the detection of P16^INK4a^ expression on tissue samples for immunohistochemical staining. Among the five studies, three directly reported the HR values [1, 9, 11], and HRs with 95% CIs of the other two were extracted from the K-M curves [10, 12]. The mean or median follow-up time among these studies ranged from 1.8 to 3.9 years.

### 3.2. Quantitative Synthesis Results

**Figure 4:**
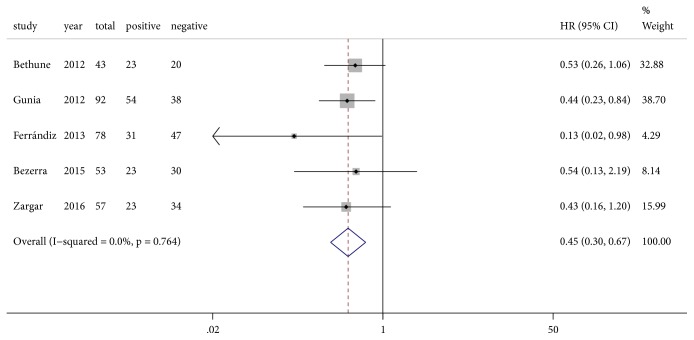
Forest plot of the pooled outcome of five eligible studies on the association between P16^INK4a^ and penile cancer prognosis; HR, hazard ratio; CI, confidence interval.

**Figure 5:**
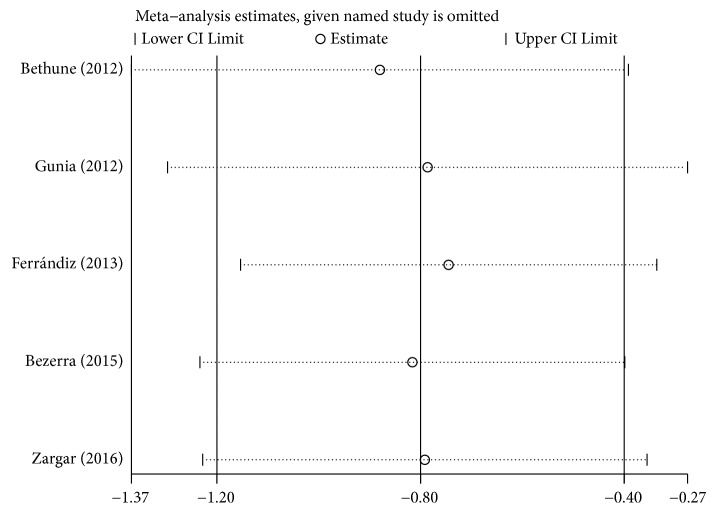
Sensitivity analysis for individual studies on the association between P16^INK4a^ and penile cancer prognosis.

**Figure 6:**
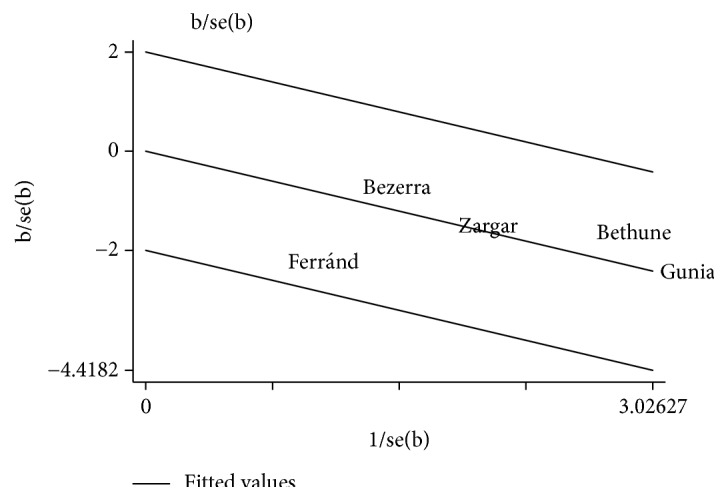
Galbraith plot of individual studies on the association between P16^INK4a^ and penile cancer prognosis.

We found no heterogeneity among the included studies (I^2^ = 0%, P=0.764) (Figure 4), which was also confirmed by sensitivity analysis and Galbraith plot (Figures 5 and 6). Survival outcomes of the five eligible studies were performed on a fixed-effects model, and we found that P16^INK4a^ positive expression was significantly associated with a better CSS of penile SCC (HR=0.45, 95%CI: 0.30-0.67, P<0.001).

**Figure 7:**
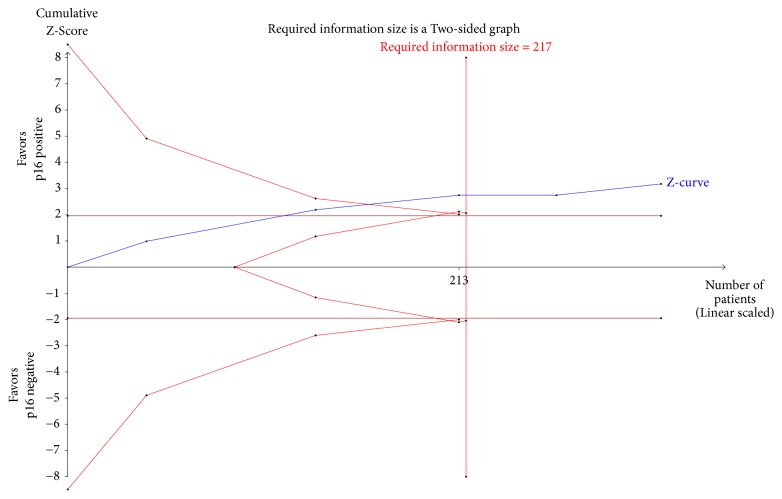
Trial sequential analysis based on the publication year of the five included studies.

A random-effects model (DL) was applied in the TSA for the five enrolled studies. The heterogeneity-adjusted required information size to demonstrate or reject a 54.3 % relative risk reduction (low-bias risk trial estimate) of P16^INK4a^ positive patients (with *α* value of 5% and a *β* value of 20%) was 217 (Figure 7). The cumulative Z-curve crossed both the conventional boundary for benefit and the required information size, indicating that the evidence was sufficiently conclusive and no further trials were required.

### 3.3. Publication Bias

**Figure 8:**
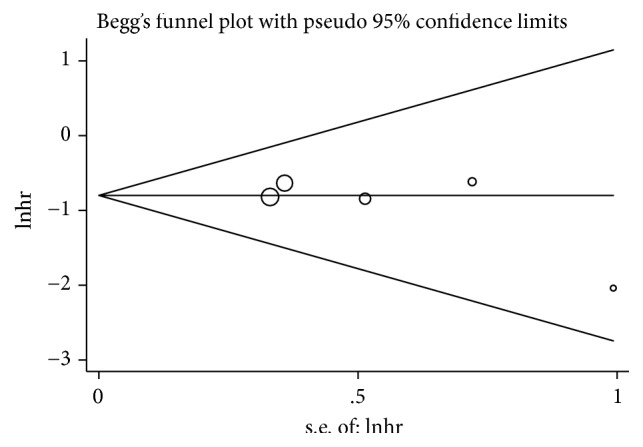
Begg's funnel plots utilized to assess publication bias.

Begg's test and Egger's test were, respectively, conducted to evaluate the publication bias. As expected, the funnel plot of Begg's test exhibited symmetricalness with a P value of 0.462, and the P value of Egger's test was 0.249 (Figure 8). Therefore, no evidence of publication bias in this meta-analysis was observed.

## 4. Discussion

As a cancer of male sex organ, penile cancer and its treatment can seriously impact sexuality and intimacy, body image, urinary function, mental health, and life quality [19]. Historically (partial) penectomy and penile sparing were useful surgical treatment of penile cancer; however, there remained a potential possibility of recurrence. Therefore, a reliable biomarker that can predict survival outcome for penile cancer is urgently required. Alterations in the P16/CyclinD1/Rb and ARF/Mdm2/P53 pathways are frequent events in the pathogenesis of SCC, especially the upregulation of P16^INK4a^ at an early stage of tumorigenesis [20]. P16^INK4a^ induces cell cycle arrest and prevents cell division by inhibition of CDK4 and CDK6 and inhibition of CDK-mediated phosphorylation of the Rb gene [21]. Positive and negative regulation of tumor suppressor P16^INK4a^ expression has been described in penile carcinoma [22], and thus the mechanisms and the predictive value of P16^INK4a^ in penile SCC requires further investigation.

Our meta-analysis found that P16^INK4a^ was an independent prognostic factor for CSS in penile SCC. In the TSA, the pooled sample size was 323, which has surpassed the required information size of 217. Besides, the cumulative Z-curve has already crossed the conventional boundary and the *α*-spending monitoring boundary, suggesting that our meta-analysis was of sufficient evidence. In addition, there existed several advanced points in our meta-analysis. First, we strictly followed the inclusion criteria for articles, so as to ensure the quality of enrolled literatures. Second, the pooled sample size of our study was larger than that of any individual study, making the effect estimation more precise. Third, there was no statistical heterogeneity among the included studies (I^2^ = 0%, P=0.764), and thus a fixed-effects model was utilized. Fourth, the pooled HR of P16^INK4a^ for CSS was 0.45, which was statistically significant (P<0.001) and was strong enough. Empirically, a predictive factor is considered to be strong when the value of HR is less than 0.5 or more than 2.0 [23]. Moreover, no publication bias was observed in this meta-analysis.

However, several limitations remained to be further refined. First of all, there were five eligible articles included in quantitative estimation, which led to the insufficiency of studies for subgroup analysis. Besides, there was no individual study on Asians, which might cause potential selection bias that hinders the comprehensive investigation of the association between P16^INK4a^ and penile SCC. Secondly, different researchers of included studies applied different cut-off values due to the lack of uniform standard for P16^INK4a^ expression. This might affect the effectiveness of P16^INK4a^ as a predictive factor for prognosis of penile SCC. Thirdly, although the pooled outcome of the five included studies indicated that P16^INK4a^ positive expression was significantly associated with a superior CSS in penile SCC (P<0.001), individual results of three studies among them showed no statistical significance. Therefore, the prognostic value of P16^INK4a^ for Penile SCC remained to be further investigated for confirmation [1, 11, 12]. Fourthly, several pathological characteristics of patients with penile SCC, such as tumor stage, differed among different studies. For instance, Ferrándiz et al. enrolled 24 cases of tumor stage I and 39 cases of tumor stage >I for original research [10]. However, the study of Bethune et al., respectively, included 24 patients of tumor stage I, 16 of tumor stage II, and 3 of tumor stage III [1]. In addition, several drawbacks such as different median or mean follow-up times among included studies were observed.

In conclusion, P16^INK4a^ positive expression is independently associated with improved CSS for patients with penile SCC, and TSA has been conducted for the first time to assess the quantity and strength of current evidence. In addition, a number of studies, which focus on the relationship between P16^INK4a^ and penile cancer prognosis in Asian population, are required for further investigation in the future.

## Figures and Tables

**Table 1:** Main characteristics of included studies.

First author & Study year	Nationality	Age (yrs)	P16^INK4a^ positive (n)	P16^INK4a^ negative (n)	Detection method	Cut-off value	Follow-up time (yrs)	HR	95% CI
Bethune 2012	Canada	63.0 mean	23	20	IHC	>0	3.9 mean	0.53^∧^	0.26–1.06
Gunia 2012	Germany	67.2 mean	54	38	IHC	>0	2.7 median	0.44^∧^	0.23–0.84
Ferrándiz 2013	Spain	69.0 median	31	47	IHC	H-score: 50	2.5 median	0.13^*∗*^	0.02–0.98
Bezerra 2015	United States	65.0 median	23	30	IHC	NM	NM	0.54^∧^	0.13–2.19
Zargar 2016	United States	60.0 median	23	34	IHC	Median	1.8 median	0.43^*∗*^	0.16–1.20

n, number; IHC, immunohistochemistry; yrs, years; NM, not mentioned; H-score, histological score; HR, hazard ratio; CI, confidence interval;

∧, HR outcome directly reported by studies; ∗, HR outcome extracted from survival curve.

## Data Availability

The hazard ratio data and trial sequential analysis data used to support the findings of this study are included within the article. The data supporting this meta-analysis are from previously reported studies and datasets, which have been cited.
